# An Explainable Deep Learning Approach for Stress Detection in Wearable Sensor Measurements

**DOI:** 10.3390/s24165085

**Published:** 2024-08-06

**Authors:** Martin Karl Moser, Maximilian Ehrhart, Bernd Resch

**Affiliations:** 1 Department of Geoinformatics, University of Salzburg, 5020 Salzburg, Austria; maximilian.ehrhart@plus.ac.at; 2 Center for Geographic Analysis, Harvard University, Cambridge, MA 02138, USA; bresch@fas.harvard.edu

**Keywords:** stress detection, deep learning, explainable AI, LSTM, Deep Generative Ensemble, generative adversarial network, integrated gradients, physiological sensor data, wearable sensors

## Abstract

Stress has various impacts on the health of human beings. Recent success in wearable sensor development, combined with advancements in deep learning to automatically detect features from raw data, opens several interesting applications related to detecting emotional states. Being able to accurately detect stress-related emotional arousal in an acute setting can positively impact the imminent health status of humans, i.e., through avoiding dangerous locations in an urban traffic setting. This work proposes an explainable deep learning methodology for the automatic detection of stress in physiological sensor data, recorded through a non-invasive wearable sensor device, the Empatica E4 wristband. We propose a Long-Short Term-Memory (LSTM) network, extended through a Deep Generative Ensemble of conditional GANs (LSTM DGE), to deal with the low data regime of sparsely labeled sensor measurements. As explainability is often a main concern of deep learning models, we leverage Integrated Gradients (IG) to highlight the most essential features used by the model for prediction and to compare the results to state-of-the-art expert-based stress-detection methodologies in terms of precision, recall, and interpretability. The results show that our LSTM DGE outperforms the state-of-the-art algorithm by 3 percentage points in terms of recall, and 7.18 percentage points in terms of precision. More importantly, through the use of Integrated Gradients as a layer of explainability, we show that there is a strong overlap between model-derived stress features for electrodermal activity and existing literature, which current state-of-the-art stress detection systems in medical research and psychology are based on.

## 1. Introduction

Stress, whether in the form of short- or long-term exposure, poses major health threats for society [1,2,3,4]. Short-term, or acute, stress can lead to instantaneous health hazards such as accidents caused by poor driving decisions [5], whereas long-term, or chronic stress, can cause serious mental conditions affecting people’s physical and psychological health [2].

Wearable-based physiological data collection has become a main line of research with applications in the fields of medicine, psychology, bioinformatics, and geoinformatics. Due to the advancement of sensor technologies, several biosignals have been used to derive and understand the complex psychophysiological response of the human body in accordance with a particular environmental or psychological stressor [6]. Before individual sensor recordings can be leveraged by an algorithm, appropriate signal filtering and processing procedures need to be performed to remove noise and other unwanted artifacts from the signal. After preprocessing the raw data, biometric features can be derived from the time- and frequency-domain representation of the signal. These features are then used as input to expert-driven rule-based [7,8] or machine learning (ML) [9] classification systems for the detection of stress.

While machine learning approaches have been shown to be effective approaches for identifying a (non-)linear mapping function between a set of input features and a particular output, the individual algorithms still require manually crafted features as inputs. Time-series biosignals, however, can be considered unstructured information, data without an inherent data model or a predetermined organization, where features have to be derived from aggregations of their time- or frequency-domain representations. This is where deep learning (DL) approaches have come to shine within recent years. Deep learning algorithms are known to be highly non-linear systems, which are powerful tools to learn structure from data [10]. End-to-end neural network (NN) architectures can deal with unstructured, raw data inputs and allow for skipping the manual feature-engineering step in the ML pipeline. A prominent example is the transformer architecture, which recent large language models (LLMs), e.g., BERT [11], are based on.

A major requirement for all these approaches, however, is the amount of available data that the model can be trained on. Depending on the type of input data, different NN architectures are advantageous with respect to their ability to process their respective data sources. For text and image data, there exists a vast number of open data sources that can be used to (pre-) train individual architectures. Within the domain of wearable sensor data, there are only a small number of open-source datasets, e.g., the WESAD dataset [12], which has frequently been used to detect stress and other health conditions based on the various psychological stress inducement scenarios that are used to simulate a particular stress condition, e.g., the Trier Social Stress Test (TSST) [13]. In addition to this lack of publicly available benchmark datasets for the evaluation of physiological responses, existing methodologies have focused on the long-term effects of stress, using physiological biosignal data over time periods of several minutes, hours, or even days [6,14,15], limiting the comparability of the proposed methodologies’ results.

Considering that there are sufficient training data available, another downside of the increased predictive capabilities of complex DL models is the lack of explainability. Feature extraction mechanisms in the form of multiple non-linearities make the interpretation of the decision process of the algorithm non-trivial, constituting a major drawback of several NN architectures. This lack of explainability in artificial intelligence (AI) methods has increasingly drawn attention to a new field of research, explainable AI (XAI). The main goal of XAI methods is to uncover the hidden processes involved in feature learning and decision-making, with the aim of better understanding the algorithmic decisions and making results more interpretable. The central motivations of explainable AI approaches are the explainability of a model’s decision process, the interpretability of the model’s predictions, and the transferability of the resulting information to other problem settings. A popular class of XAI techniques is feature attribution, e.g., through Integrated Gradients (IG) [16], which aims to find the most relevant features used by the model to arrive at a particular prediction [17]. Within the context of stress detection based on psychophysiological reactions, the main objective is to automatically derive meaningful and explainable features from subsequences of a time series, ideally relating subjective human stress responses to existing knowledge stated in current literature.

Taking into consideration these challenges, we propose a deep learning framework to detect acute stress in a time window of 16 s, focusing on the explainable derivation of physiological signal features that indicate an immediate response to a stress stimulus. Potential applications of the proposed methodology range from traffic and urban planning use cases to medical research. Within the context of urban planning, deriving stress from psychophysiological reactions of the human body, captured by non-invasive wearable sensor technology, can assist city planners in better understanding people’s subjective perceptions at different road segments and intersections. An example application in the field of medicine would be to evaluate the effectiveness of anesthesia based on people’s perceived stress levels. The proposed algorithm is evaluated based on a dataset that was recorded in a strictly controlled laboratory environment where audio stress stimuli were used to induce states of emotional arousal.

The main contributions of our work are as follows:

- An ensemble of LSTM networks, enriched through a Deep Generative Ensemble of conditional GANs [18], outperforms current state-of-the-art rule-based stress detection systems proposed by [7,8] by 3 percentage points in terms of recall and 7.18 percentage points in terms of precision.
- Integrated Gradients offers an XAI approach to highlight the significant features used by the DL model to predict stress. For electrodermal activity, these features are in line with existing literature and expert knowledge.
- Skin temperature does not lead to significant contributions in the classification of acute stress, neither in the rule-based system nor in the DL approach.
- DL methodologies enable the automatic derivation of meaningful features from raw physiological biosignals in the time and frequency domains.

## 2. Related Work

Researchers in the field of physiological stress detection have proposed several methods to automatically classify stress-related events. Within this process, various use cases, distinct physiological indicators, and different experimental protocols were tested to classify stress in human subjects [6,19]. Some of the most promising physiological signals related to stress are electrodermal activity (EDA), skin temperature (ST), and cardiovascular activity measurements such as heart rate (HR), heart rate variability (HRV), and inter-beat interval (IBI) [6,20].

The current literature shows that EDA and derived features serve as especially reliable indicators for the detection of instant and non-subjective states of emotional arousal [6,15,21,22]. Based on a bandpass frequency filter, the raw EDA signal can be split up into the Skin Conductance Level (SCL), also known as the tonic part, and the Skin Conductance Response (SCR), which describes the phasic part of the signal, where non-specific SCR can be mainly attributed to noise [6].

The SCR component of the EDA signal correlates with immediate stress, showing an immediate increase in the signal component as a response to a stressor [23]. It has been shown that EDA, in combination with the cvxEDA [24] preprocessing methodology and a support vector machine, is sufficient to accurately classify stress [22]. A study conducted by [15] shows a strong correlation of the EDA signal with stress during driving tasks, highlighting the suitability of Skin Conductance Response measurements for stress assessments in non-stationary settings.

Another biosignal marker that has frequently been attributed to stress is ST [6,25]. The findings of these studies suggest that during a stress response of the human body, ST either decreases or increases, depending on the different body part the sensor is applied to. Reference [26] states that ST significantly decreases at the hand-palm and fingertips during the Trier Social Stress Test (TSST), but it increases when measured at the upper arm. Another study [25] suggests filtering the ST signal and using the information contained in the slope instead of the mean value of the ST signal.

While previous studies propose different ST features in relation to stress, it is mainly long-term effects of ST changes in accordance with a stress response that are considered [27]. On the other hand, other studies look at the short-term response of ST and derived features, where oscillations caused by a stressor were found in the low frequency spectrum of the signal due to blood flow changes. Hence, appropriate frequency filtering enables the measurement of ST changes as a response to acute stressors.

To automatically detect stress from psychophysiological data, the majority of research leverages machine learning methods [19].

Before individual signals can be fed as input to a ML algorithm, feature engineering needs to be performed, where various features are manually extracted via summary statistics over a given sliding window [12,28,29]. The main issue here is the cost of feature engineering, where expert knowledge or extensive feature selection heuristics need to be evaluated to find valuable features that serve as indicators for stress.

Recent deep learning techniques have been shown to be successful feature extractors when exposed to raw input data, making them valuable tools for end-to-end machine learning pipelines. In these approaches, features are extracted automatically by the neural network. Ref. [30] has shown that a Convolutional Neural Network (CNN) outperforms traditional machine learning approaches on various emotion and stress classification tasks, leveraging the WESAD [12] dataset. In [28], the authors compare handcrafted features as input to ML algorithms with deep learning methods and conclude that modern DL architectures show better performance in the task of classifying stress.

While previously mentioned DL approaches have proven suitable for handling complex, unstructured input data, considering there exist a sufficient amount of training data [31], their main drawback is the lack of explainability and interpretability.

Rule-based systems, crafted based on expert knowledge, offer an attractive alternative in this case, where the decision process of the algorithm follows a logical structure and results can be communicated to stakeholders more transparently. However, to avoid issues of generalization to new test subjects, physiological differences among individual participants are important to consider [7].

Considering the strengths and weaknesses of the aforementioned approaches, we identified research gaps in terms of the explainability of deep learning methods, and the suitability of these methodologies to work as automatic feature extractors in the context of time-series physiological sensor data. To close this research gap, the main goal of this paper is to compare rule-based expert systems such as [7,8] with an explainable deep learning algorithm to evaluate the model’s ability to automatically extract highly non-linear features from physiological data sources, serving as short-term time features for the task of classifying acute stress. Explainability is added to the approach by using Integrated Gradients [16] to uncover the most relevant time-dependent features used by the DL algorithm for generating predictions.

## 3. Methodology

We propose a deep-learning-based approach for stress detection in wearable sensor data considering a short-term time window of 16 s. The results of the DL methodology are compared to state-of-the-art rule-based expert systems, where the physiological dataset used for training and evaluation was collected in a strictly controlled laboratory test setting at the University of Salzburg, Austria. To assign appropriate labels to the physiological signals at given stress times, a specifically designed experimental protocol was followed. The data collection process is further described in Section 3.1.

**Figure 1:**
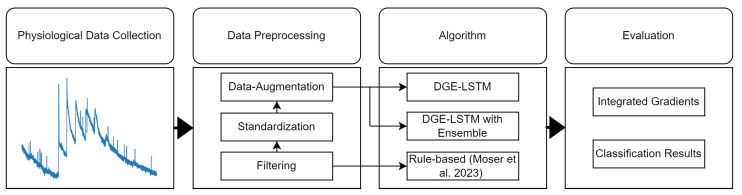
Overview of the methodology, with Rule-based algorithm based on [7].

For a comparison of the individual approaches, we developed a method that collects, processes, and then automatically classifies individual stress moments with rule-based and deep-learning-based algorithms. The results of the individual methodologies are used to evaluate the algorithms’ performance in terms of recall, precision, and accuracy, with a particular emphasis on explainable AI (XAI), concretely focusing on the interpretability of the approach. A complete description of the rule-based algorithm and the implementation details can be found in [7]. Figure 1 gives an overview of the methodology.

### 3.1. Physiological Data Collection

To the best of our knowledge, there is no publicly available dataset at the time of this writing where stress labels are given on the granularity level of individual seconds, which would be suitable for the task of short-term stress classification. To close this gap, we collect our own physiological stress datasets, where we follow the laboratory experimental protocol introduced in [7,8,32]. High-quality physiological sensor data were collected between 2018 and 2022, resulting in a dataset consisting of 28 subjects. All participants were asked to fill out a voluntary consent form, which informs participants on the purpose of the study and how the data are anonymized before further processing. Additionally, all voluntary test subjects were asked to not consume any stimulants prior to the experiment. Each subject was equipped with the Empatica E4 wristband, which was then connected to an eDiary app [33] to save the data into individual SQLite database files. More information about the sensor’s technical specifications is available at [34]. Participants were divided into groups of 5, 4, or 3 persons per session, and each session lasted between 15 to 22 min. Each volunteer participated in one group and one session only, so there are 28 different subjects who make up the dataset. After a short initiation phase to determine a baseline during a person’s state of relaxation, 10 stress-causing interventions were performed through an air horn sound, an audio stimulus that should induce a stress reaction. The air horn sounds occurred in random time intervals ranging from 52 s up to 125 s, with an average distance of 86 s between two consecutive stimuli. More details on the data collection procedure can be found in [7,8,32].

### 3.2. Signal Processing

To prepare the data for the different stress-detection methodologies, the following preprocessing steps were performed. First, individual signals obtained from the laboratory test participants were excluded when the sensor did not record any data or in the case of highly noisy recordings caused by improper sensor attachment. The resulting data therefore present a homogeneous physiological dataset of high quality that will be made available to the public to foster research in the domain of physiological stress detection. EDA and ST signals were filtered using a bandpass filter, implemented in the SciPy python package [35]. Prior to applying a first-order high-pass filter with a cutoff frequency of 0.05 Hz to extract the phasic component of the EDA signal, the raw signal was processed with a low-pass filter to remove noise. For the frequency filtering of the skin temperature signal, we use the same approach as proposed in [7,32].

After filtering both signals based on their frequency representation, each signal was downsampled from the original E4 sampling frequency of 4 Hz to 1 Hz in order to further clean the signals from artifacts that can be attributed to movement. In the next step, the signals were standardized by subtracting the mean of the signal μ from each feature value *x* and dividing by the signal’s standard deviation, σ, ($\frac{x-\mu }{\sigma }$) to have a mean of 0 and a standard deviation of 1 for each signal. The standardization of the individual signals has the effect of making the training procedure of the deep learning algorithm more efficient and removing bias related to differing value ranges among the attributes from the data, due to individual inputs being converted to the same scale. To finalize the preprocessing pipeline, the signals were split into sequences using a sliding window of 16 s, in which common EDA features such as the variation in latency between stressor and the onset of the EDA, the rising time from the onset to the peak, and the recovery of the signal to the usual state are included. The individual characteristics of the EDA response, their duration, and the time window of 16 s are chosen based on current literature [6,36].

### 3.3. Deep Learning for Physiological Stress Detection

This section explains the proposed deep learning methodology to classify acute stress in more detail. Since we use physiological time series data, a recurrent neural network architecture with a sigmoid layer at the end provides a suitable choice to extract features from the sequential input signal and perform the classification. Since we focus on the derivation of explainable features through IG, we stick to a classic recurrent neural network (RNN) architecture variant, the LSTM, rather than identifying the best possible combination of network architecture and hyperparameters for our classification setting. We decided against transformer architecture variants as we have a relatively short sequence length and our learning task does not require the identification of long-term dependencies. We opt for the LSTM network architecture as it provides a sufficiently complex recurrent neural network variant to capture the short-term time-dependent dependencies in the signal. The LSTM [37] deals with the vanishing gradient problem by using a memory cell, $c_{t}$, and different gating mechanisms, the input gate $i_{t}$, the forget gate $f_{t}$ and the output gate $o_{t}$. As shown in Equation (5) the recurrent connections in the network are set to the identity, resulting in a constant error carousel, with the effect that the error is distributed evenly among the time steps when weights are updated during backpropagation. As displayed in Equations (1)–(4), the gating mechanism controls how much information gets into and out of the memory cell by applying a nonlinear transformation function, i.e., sigmoid, to the weighted sum of the inputs $x_{t}$, the previous hidden states $h_{t-1}$, and the bias units *b*. The forget gate [38] was introduced to learn the removal of information from the memory cell, when the learning process gets saturated. Introducing the forget gate comes at the cost of re-introducing the problem of vanishing gradients, which can be mitigated by initializing the forget gate bias with 1 [37,38,39]. In Equation (6), the updated hidden state is then computed. The recurrent network connection is initialized orthogonally, the input weights with xavier, and biases are initialized with 0. Further details on the theoretical considerations of the LSTM architecture can be found in [37]. To determine the best hyperparameters for the network, a grid search with cross-validation (CV) on the training dataset is performed. The defined grid-search space can be seen in the appendix, where results of the five-fold CV are displayed in bold Table A1.

(1) $$\begin{array}{c} i_{t}=\sigma \left(W_{i}x_{t}+b_{ii}+R_{i}h_{t-1}+b_{hi}\right) \end{array}$$

(2) $$\begin{array}{c} f_{t}=\sigma \left(W_{f}x_{t}+b_{if}+R_{f}h_{t-1}+b_{hf}\right) \end{array}$$

(3) $$\begin{array}{c} z_{t}=\sigma \left(W_{z}x_{t}+b_{ig}+R_{z}h_{t-1}+b_{hg}\right) \end{array}$$

(4) $$\begin{array}{c} o_{t}=\sigma \left(W_{o}x_{t}+b_{io}+R_{o}h_{t-1}+b_{ho}\right) \end{array}$$

(5) $$\begin{array}{c} c_{t}=f_{t}⊙c_{t-1}+i_{t}⊙z_{t} \end{array}$$

(6) $$\begin{array}{c} h_{t}=o_{t}⊙tanh\left(c_{t}\right) \end{array}$$

While having a relatively large sample of test subjects compared to other studies [12], our dataset can still be considered small for training a NN. To deal with the relatively small sample size and the imbalance with regard to stress and non-stress sequences, we used the conditional GAN proposed in [32] to augment the dataset with synthetic data. To improve generalization while preventing overfitting on the training data, we added random Gaussian noise to the stress moments and scaled the amplitude randomly, ultimately arriving at new samples for the training dataset [40]. To increase generalization, we leverage the Deep Generative Ensemble (DGE) approach proposed in [18]. Within this ensemble, synthetic data are generated from different random seeds, where the predictions of the classifiers are then averaged for each of the seeds. This mitigates the effect of overfitting to the generative model’s samples, resulting in better predictions within a low-data regime. To reduce variations in the predictions caused by a small test dataset, a homogeneous ensemble [41] of LSTMs is used as our final classification model. The LSTM architecture is implemented with PyTorch version 1.13.1 [42].

**Figure 2:**
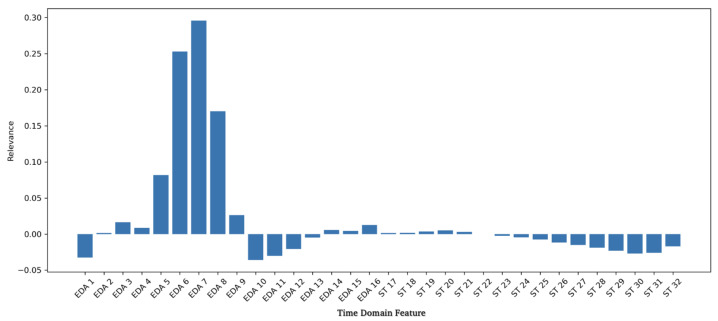
The feature relevance to stress from one seed of the ensemble. Time-domain features are shown on the x-axis with references to the certain time-step.

On top of the DL-based classification model, we apply Integrated Gradients (IG) [16] to identify important features derived by the model with regard to classifying sequences as stress. In this time-series setting, we compute a straight-line path integral from a baseline, i.e., zero vector, to the original input over which the gradients at each point are calculated. As IG is a local feature attribution method, we take the average over all the stress samples in the test set to derive global information concerning the features used for predicting stress. Figure 2 displays the relevance of the derived time-domain features in terms of stress predictions for one selected seed and ensemble model, averaged over all participants. IG values are subtracted from the baseline, where positive and negative relevance scores are constructed, highlighting the importance of individual time steps in the binary stress classification setting.

## 4. Experiments and Results

To compare the rule-based system proposed in [7] with the deep learning algorithm described in the previous section, a number of different experiments were carried out. The first experiment focuses on the classification performance of the algorithms on our test dataset, using different seeds in the train–test split. In the second experiment, contributions of the individual features with regard to the overall classification performance are tested. The third and final experiment uses Integrated Gradients to enhance the interpretability and explainability of the results produced by the DL algorithm. The test dataset for each of the experiments consists of 10 randomly selected participants from the study setup described in Section 3.1.

### 4.1. Stress Detection Results

The first experiment shows the ability of the LSTM and the rule-based algorithm to classify stress in a short-term time window of 16 s. Among the 28 laboratory test subjects, we performed a train–test split on the participant level, randomly selecting 10 participants per seed for evaluation and the remaining 18 participants for training. The LSTM network is reinitialized in each of the three seeds and the training procedure is terminated once the validation loss stops improving. As mentioned in Section 3.3, the best hyperparameter setting is determined based on a training dataset selected through five-fold cross-validation. The selection is based on the F1 score, with a special focus on recall, to avoid any False Negative (FN) predictions, corresponding to missed reference stress moments in our classification task. The hyperparameter setting with the maximum F1 score is chosen as the final model. For the rule-based algorithm, we implement and use the same rules and parameters as [7]. Each algorithm is evaluated based on a sliding window approach, where a specified sliding window is moved over all participants within the evaluation set.

**Figure 3:**
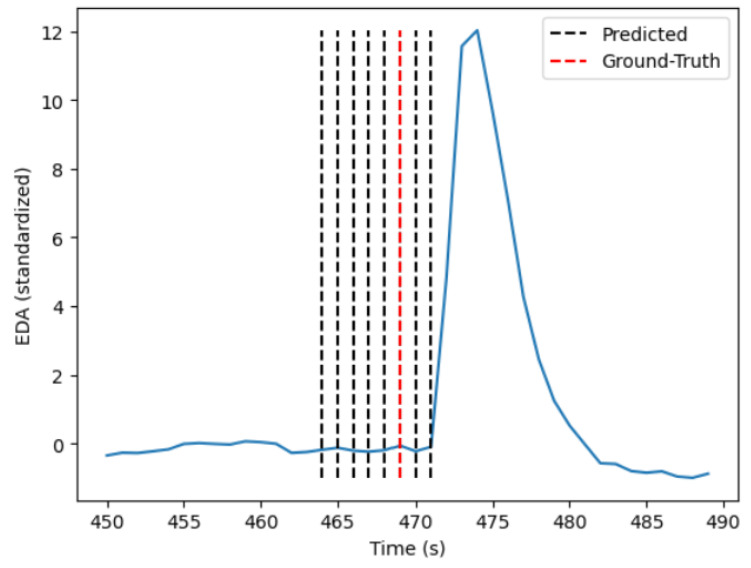
A moment of stress and the predictions over various sliding windows.

A True Positive (TP), a detected stress moment that coincides with a reference stress moment, is considered when the algorithm’s prediction is within a time window of 2 s before and 6 s after a reference stressor. This evaluation window was chosen to account for all the variations within a stress situation considering the current EDA literature [6,36]. A False Positive (FP) is a moment of stress (MOS) predicted by the algorithm, where, within an 8 s time window, no reference stressor occurred. On the contrary, if there is a reference stress moment and no prediction from the algorithm within 8 s, then the prediction is considered a False Negative (FN). Lastly, if the algorithm does not predict a stress situation and within 8 s of this prediction there is also no stressor, then the prediction is considered a True Negative (TN). To avoid an artificially high number of TPs and TNs, predictions that occur within a series of 8 s are summarized into one stress prediction, as can be seen in Figure 3.

Table 1 shows the results of the stress detection experiment. The best average recall is achieved by the LSTM (DGE) with an average score of 0.7633, followed by the ensemble of LSTMs (DGE), with an average recall score of 0.7367, and the rule-based algorithm with an average recall score of 0.7333. Precision values are higher for the deep learning approaches, with an average precision score of 0.384 for the LSTM ensemble (DGE) and an average score of 0.359 for the LSTM (DGE). The rule-based algorithm reaches an average precision of 0.323, which is significantly lower than the ones achieved by the proposed DL models. In terms of accuracy, the best average score stems from the ensemble LSTM (DGE), with an accuracy of 0.9816, followed by the LSTM (DGE) with an accuracy of 0.9809, and the rule-based algorithm, with an accuracy score of 0.9806. The best recall on a single seed, however, is from the rule-based algorithm, with a maximum score of 0.82.

### 4.2. Results with Regard to ST Contribution

The results of the second experiment show the contributions of the EDA and the ST signal to the overall classification score of the LSTM, considering the test dataset. The LSTM model is trained without data augmentation, but with the same hyperparameters determined by the previous experiment Section 4.1, which can be seen in the appendix. Similarly, the same test dataset seeds are used for evaluation. Table 2 displays the results of the experiment on three different train–test split seeds. The first column shows the results of training with the EDA signal only, whereas the second shows the results of a combination of both signals during the training process. The average recall value with the combination of EDA and ST exceeds the one from EDA by an average of 2.66%. While this suggests that adding the ST signal to the model enhances the algorithm’s stress prediction capability, this result should be interpreted with care, as the improvement mainly comes from one seed, hinting towards significant differences within the test individuals’ stress reaction, expressed in the form of skin temperature variations. In terms of precision, there is only a minor average difference of 0.12% between the EDA and the combination of the two.

### 4.3. Interpretability of the Deep Learning Approach

As stated in the literature [6,8,36] and used in the rule-based system [7,8], the relevant features of the phasic part of the EDA signal are the rise time from the onset to the peak, the latency from the stressor to the onset, and the recovery time of the signal back to the usual state. This is also present in the features used by the DL model. Figure 2 highlights that the most relevant features are the rise of the peak and the recovery of the signal after the peak. Concerning the ST signal, where immediate patterns are not as clear as in the phasic component of the EDA signal, the model only found little relevance in the initial time frame of the signal and even some negative contributions toward the end of the signal. This can be interpreted as the likelihood of stress predictions being reduced if the given features are present and used by the model.

## 5. Discussion

### 5.1. Discussion of Methodology

The paper proposes a methodology to classify short-term stress-related events with an ensemble of LSTMs, enriched through a Deep Generative Conditional GAN, and compares the results to a state-of-the-art rule-based system [7].

As already mentioned, a deep learning approach tends to require plenty of training data to generalize well on unseen data [31]. Since the collection of a physiological dataset with ground-truth labels is costly, our dataset, containing 180 MOS for training and 100 MOS for testing, ends up being relatively small for a DL setting. To mitigate this problem, data-augmentation and a deep ensemble are used to reduce the chance of overfitting and minimize the variance within the predictions. We would recommend a similar procedure for researchers who also work in the low data regime, e.g., [12].

As generative models such as the conditional GAN [32] used in this work suffer from problems such as the memorization of the dataset, mode collapse, and noisy data, we decided to use the Deep Generative Ensemble (DEG) approach proposed by [18]. It has been shown that, especially in the low-data regime, there is an improvement with the DGE approach. While this approach helps with typical problems that generative models face, it comes at the cost of increased computational complexity, as the generation process needs to run multiple times. Since we are dealing with a relatively small dataset, DGE provides a feasible approach for our use case.

We decided to use a simple LSTM network, since we wanted to demonstrate the capabilities of a deep learning algorithm to automatically extract meaningful and interpretable features from sequential physiological signals. To further improve the classification performance of the model, a more powerful deep ensemble method, e.g., the stacking of a more heterogeneous ensemble of different model classes or hyperparameters [41], can be tested. In future studies, we suggest trialing other backbone architectures, e.g., a CNN or transformer, in combination with feature attribution methods such as Integrated Gradients, and comparing results to our presented algorithm.

To test the generalization capabilities of the presented algorithm, additional data collection studies in a laboratory test setting or non-stationary, real-world environments should be considered. One could use uncertainty estimation, as proposed in [43], to perform out-of-distribution classification and test the generalization capabilities of the model on different datasets.

### 5.2. Discussion of Results

The LSTM with DGE proposed in this paper outperforms the rule-based algorithm in terms of average recall and average precision.

The rule-based algorithm achieves an average value of 0.7333 and 0.2872 in terms of recall and precision. The LSTM with DGE results in an average recall of 0.7633 and an average precision of 0.359, demonstrating the capability of the DL methodology to derive meaningful features from the raw signal data.

**Figure 4:**
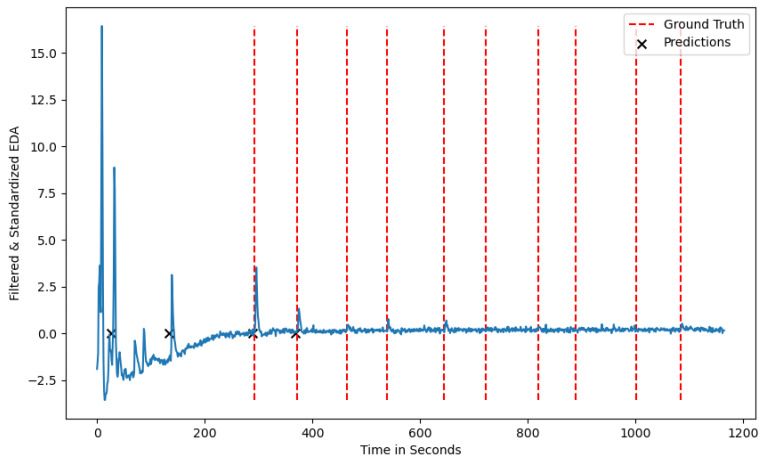
A test participant from the test dataset with the ground-truth stressors and the predicted stressors without a reaction at the ground-truth labels.

Both algorithms show some variations in terms of performance metrics for the different seeds. This variation can have several causes, one of them being that certain participants show a less intense physiological reaction than others. Familiarity with a stress stimulus that is induced multiple times could also lead to less prominent physiological reactions and, in extreme cases, to no reaction at all. The decreased stress response pattern of one participant can be seen in Figure 4, where only 4 out of 10 ground-truth stressors are visually present in the signal. Considering this observation, no algorithm would be able to detect more than the number of stress responses inherent in the phasic component of the EDA signal, leading to a decreased recall score for certain seeds.

**Figure 5:**
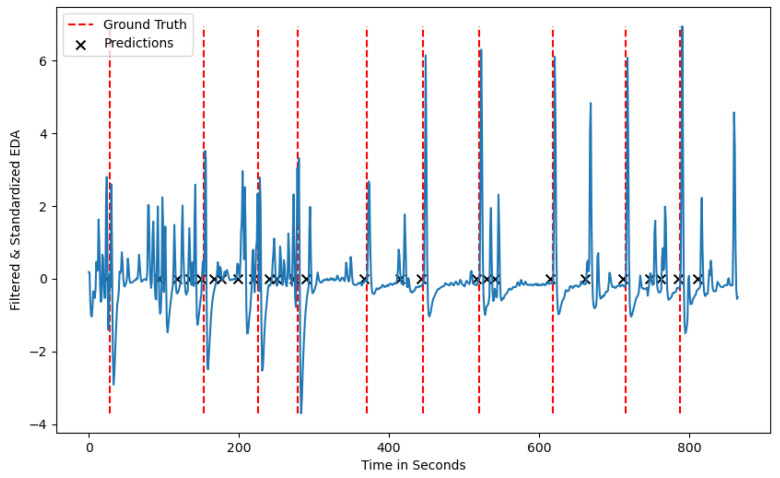
A test participant from the test-dataset with the ground-truth stressors and the predicted stressors showing noise in between the ground-truth labels.

Low average precision values can be attributed to participants who show phasic EDA peaks between the ground-truth stressors, which the algorithm considers as stress situations. This is visualized in Figure 5, where multiple peaks occur between the induced stress times and can be attributed to participants being stressed by other sources or the sensor being loosely attached to a participant’s wrist. Depending on the use case the algorithm is applied to, the trade-off between TP and FP needs to be considered. As we plan on using the algorithm in the context of urban planning to detect spatio-temporal stress clusters, we aim for a high recall value that minimizes the number of FN predictions, i.e., stress situations the algorithm fails to detect [44]. To further reduce the number of FNs, we aim to incorporate other biometric information such as cardiovascular activity and eye-tracking data. Adding such information could lead to a more comprehensive understanding of the situation and potential stress-causing factors.

## 6. Conclusions

We propose an explainable LSTM approach to acute stress classification in a controlled laboratory environment based on wearable sensor data, where we compare the results to a state-of-the-art rule-based system that implements domain knowledge from experts. The proposed deep LSTM ensemble method for detecting stress improves the current state of the art by an average recall of up to 3% and an average precision of up to 3.6%. The problem of a small and unbalanced dataset is mitigated by a combination of data augmentation, synthetic data generation based on a conditional GAN, and a Deep Generative Ensemble. To address the explainability aspects of the proposed LSTM ensemble, we use Integrated Gradients (IG) to explore the prediction behavior of the model. By using IG, we show that the LSTM learns and uses similar signal patterns as the rule-based algorithm to predict stress-related events, which is in line with the current literature on physiological stress detection. In a separate experiment, we show that the addition of the filtered short-term skin temperature signal does not significantly improve the predictive score in terms of recall and precision, implying that more research is needed to find consistent patterns in the ST signal, which can be related to acute stress.

In order to better understand and algorithmically capture the complex stress reaction of the human body, we recommend that other biomarkers that can be measured through non-invasive wearable devices, e.g., blood volume pressure (BVP), heart rate (HR), or heart rate variability (HRV), should be considered and integrated in future research.

## Figures and Tables

**Table 1:** Classification results of the different models, where K is the number of ensembles used.

	Seed	Recall	Precision	Accuracy
LSTM (DGE K = 5)	I	0.68	0.3477	0.983
	II	0.8	0.4	0.9817
	III	0.81	0.3378	0.9779
LSTM (DGE K = 5) & Ensemble	I	0.67	0.3939	0.9848
	II	0.79	0.4108	0.9824
	III	0.75	0.3475	0.9799
Rule-Based [7] (Moser et al., 2023)	I	0.64	0.3120	0.9822
	II	0.82	0.3548	0.9822
	III	0.74	0.3023	0.9799

**Table 2:** Results from the LSTM without DGE and without an Ensemble, using EDA only and then EDA and ST.

Seed	Recall	Precision	Accuracy
EDA			
I	0.53	0.3987	0.9873
II	0.66	0.4492	0.9867
III	0.63	0.363	0.9832
EDA & ST			
I	0.54	0.3761	0.9857
II	0.73	0.4349	0.9845
III	0.63	0.4033	0.9851

## Data Availability

Data are contained within the article.

## Abbreviations

The following abbreviations are used in this manuscript:

AC	Alternating Current
ANS	Autonomic Nervous System
BVP	Blood Volume Pressure
DC	Direct Current
DL	Deep Learning
ECG	Electrocardiography
EDA	Electrodermal Activity
EDL	Electrodermal Level
EDR	Electrodermal Response
FN	False Negative
FP	False Positive
FPR	False Positive Rate
GAN	Generative Adversarial Network
GNSS	Global Navigation Satellite System
GSR	Galvanic Skin Response
HR	Heart Rate
HRV	Heart Rate Variability
Hz	Hertz
IBI	Inter-Beat Interval
IG	Integrated Gradients
LDA	Linear Discriminant Analysis
LUCCK	Learning Using Concave and Convex Kernels
LSTM	Long-Short Term Memory Network
ML	Machine Learning
MOS	Moment of Stress
NN	Neural Network
PPG	Photoplethysmography
SC	Skin Conductance
SCL	Skin Conductance Level
SCR	Skin Conductance Response
ST	Skin Temperature
SVM	Support Vector Machine
TN	True Negative
TP	True Positive
TPR	True Positive Rate
VR	Virtual Reality

## Appendix A. Hyperparameters

**Table A1:** Hyperparameters tested in the Grid-Search (values in bold highlight the best hyperparameters found).

Hyperparameter	Values
Number of Layers	[1, **2**]
Number of MOS-Augmented	[**400**, 800, 1200]
Number of non-MOS-Augmented	[400, **800**, 1200]
Units	[**32**, 64]
Inital Learning Rate	[**0.01**, 0.001, 0.0001]
Learning Rate Schedular	**Cosine Scheduler**
Optimizer	**Adam with Weight Decay**

## References

[1] Hefez A., Metz L., Lavie P. (1987). Long-term effects of extreme situational stress on sleep and dreaming. Am. J. Psychiatry.

[2] McGonagle K.A., Kessler R.C. (1990). Chronic stress, acute stress, and depressive symptoms. Am. J. Community Psychol..

[3] Schubert C., Lambertz M., Nelesen R., Bardwell W., Choi J.B., Dimsdale J. (2009). Effects of stress on heart rate complexity—A comparison between short-term and chronic stress. Biol. Psychol..

[4] Dhabhar F.S. (2014). Effects of stress on immune function: The good, the bad, and the beautiful. Immunol. Res..

[5] McMurray L. (1970). Emotional stress and driving performance: The effect of divorce. Behav. Res. Highw. Saf..

[6] Giannakakis G., Grigoriadis D., Giannakaki K., Simantiraki O., Roniotis A., Tsiknakis M. (2019). Review on psychological stress detection using biosignals. IEEE Trans. Affect. Comput..

[7] Moser M.K., Resch B., Ehrhart M. (2023). An Individual-oriented Algorithm for Stress Detection in Wearable Sensor Measurements. IEEE Sens. J..

[8] Kyriakou K., Resch B., Sagl G., Petutschnig A., Werner C., Niederseer D., Liedlgruber M., Wilhelm F., Osborne T., Pykett J. (2019). Detecting moments of stress from measurements of wearable physiological sensors. Sensors.

[9] Gedam S., Paul S. (2021). A review on mental stress detection using wearable sensors and machine learning techniques. IEEE Access.

[10] Goodfellow I., Bengio Y., Courville A. (2016). Deep Learning.

[11] Devlin J., Chang M.W., Lee K., Toutanova K. (2018). Bert: Pre-training of deep bidirectional transformers for language understanding. arXiv.

[12] Schmidt P., Reiss A., Duerichen R., Marberger C., Van Laerhoven K. Introducing wesad, a multimodal dataset for wearable stress and affect detection. Proceedings of the 20th ACM International Conference on Multimodal Interaction.

[13] Kirschbaum C., Pirke K.M., Hellhammer D.H. (1993). The ‘Trier Social Stress Test’—A tool for investigating psychobiological stress responses in a laboratory setting. Neuropsychobiology.

[14] Setz C., Arnrich B., Schumm J., La Marca R., Tröster G., Ehlert U. (2009). Discriminating stress from cognitive load using a wearable EDA device. IEEE Trans. Inf. Technol. Biomed..

[15] Healey J.A., Picard R.W. (2005). Detecting stress during real-world driving tasks using physiological sensors. IEEE Trans. Intell. Transp. Syst..

[16] Sundararajan M., Taly A., Yan Q. Axiomatic attribution for deep networks. Proceedings of the International Conference on Machine Learning PMLR.

[17] La Rosa B., Blasilli G., Bourqui R., Auber D., Santucci G., Capobianco R., Bertini E., Giot R., Angelini M. (2023). State of the art of visual analytics for explainable deep learning. Comput. Graph. Forum.

[18] van Breugel B., Qian Z., van der Schaar M. (2023). Synthetic data, real errors: How (not) to publish and use synthetic data. arXiv.

[19] Vos G., Trinh K., Sarnyai Z., Azghadi M.R. (2023). Generalizable machine learning for stress monitoring from wearable devices: A systematic literature review. Int. J. Med. Inform..

[20] Kreibig S.D. (2010). Autonomic nervous system activity in emotion: A review. Biol. Psychol..

[21] Farrow T.F., Johnson N.K., Hunter M.D., Barker A.T., Wilkinson I.D., Woodruff P.W. (2013). Neural correlates of the behavioral-autonomic interaction response to potentially threatening stimuli. Front. Hum. Neurosci..

[22] Greco A., Valenza G., Lazaro J., Garzon-Rey J.M., Aguilo J., De-la Camara C., Bailon R., Scilingo E.P. (2021). Acute stress state classification based on electrodermal activity modeling. IEEE Trans. Affect. Comput..

[23] Dawson M.E., Schell A.M., Filion D.L. (2007). The electrodermal system. Handbook of Psychophysiology.

[24] Greco A., Valenza G., Lanata A., Scilingo E.P., Citi L. (2015). cvxEDA: A convex optimization approach to electrodermal activity processing. IEEE Trans. Biomed. Eng..

[25] Zhai J., Barreto A. (2006). Stress detection in computer users based on digital signal processing of noninvasive physiological variables. Proceedings of the 2006 International Conference of the IEEE Engineering in Medicine and Biology Society.

[26] Vinkers C.H., Penning R., Hellhammer J., Verster J.C., Klaessens J.H., Olivier B., Kalkman C.J. (2013). The effect of stress on core and peripheral body temperature in humans. Stress.

[27] Shusterman V., Anderson K.P., Barnea O. (1997). Spontaneous skin temperature oscillations in normal human subjects. Am. J.-Physiol.-Regul. Integr. Comp. Physiol..

[28] Bobade P., Vani M. (2020). Stress detection with machine learning and deep learning using multimodal physiological data. Proceedings of the 2020 Second International Conference on Inventive Research in Computing Applications (ICIRCA).

[29] Smets E., Casale P., Großekathöfer U., Lamichhane B., De Raedt W., Bogaerts K., Van Diest I., Van Hoof C. (2016). Comparison of machine learning techniques for psychophysiological stress detection. Pervasive Computing Paradigms for Mental Health, Proceedings of the 5th International Conference, MindCare 2015, Milan, Italy, 24–25 September 2015.

[30] Li R., Liu Z. (2020). Stress detection using deep neural networks. BMC Med. Inform. Decis. Mak..

[31] LeCun Y., Bengio Y., Hinton G. (2015). Deep learning. Nature.

[32] Ehrhart M., Resch B., Havas C., Niederseer D. (2022). A Conditional GAN for Generating Time Series Data for Stress Detection in Wearable Physiological Sensor Data. Sensors.

[33] Petutschnig A., Reichel S., Měchurová K., Resch B. (2022). An eDiary App Approach for collecting physiological Sensor Data from Wearables together with subjective observations and emotions. Sensors.

[34] E4 Wristband|Real-Time Physiological Signals|Wearable PPG, EDA, Temperature, Motion Sensors. https://support.empatica.com/hc/en-us/articles/202581999-E4-wristband-technical-specifications.

[35] Virtanen P., Gommers R., Oliphant T.E., Haberland M., Reddy T., Cournapeau D., Burovski E., Peterson P., Weckesser W., Bright J. (2020). SciPy 1.0: Fundamental Algorithms for Scientific Computing in Python. Nat. Methods.

[36] Boucsein W. (2012). Electrodermal Activity.

[37] Hochreiter S., Schmidhuber J. (1997). Long short-term memory. Neural Comput..

[38] Gers F.A., Schmidhuber J., Cummins F. (2000). Learning to forget: Continual prediction with LSTM. Neural Comput..

[39] Jozefowicz R., Zaremba W., Sutskever I. An empirical exploration of recurrent network architectures. Proceedings of the International Conference on Machine Learning, PMLR.

[40] Um T.T., Pfister F.M.J., Pichler D., Endo S., Lang M., Hirche S., Fietzek U., Kulić D. Data augmentation of wearable sensor data for parkinson’s disease monitoring using convolutional neural networks. Proceedings of the 19th ACM International Conference on Multimodal Interaction, ACM, ICMI’17.

[41] Ganaie M., Hu M., Malik A., Tanveer M., Suganthan P. (2022). Ensemble deep learning: A review. Eng. Appl. Artif. Intell..

[42] Paszke A., Gross S., Massa F., Lerer A., Bradbury J., Chanan G., Killeen T., Lin Z., Gimelshein N., Antiga L. (2019). PyTorch: An Imperative Style, High-Performance Deep Learning Library. Advances in Neural Information Processing Systems 32.

[43] Lakshminarayanan B., Pritzel A., Blundell C. Simple and scalable predictive uncertainty estimation using deep ensembles. Proceedings of the 31st Conference on Neural Information Processing Systems (NIPS 2017).

[44] Kyriakou K., Resch B. (2019). Spatial analysis of moments of stress derived from wearable sensor data. Adv. Cartogr. Gisci. ICA.

